# A 10-m resolution impervious surface area map for the greater Mekong subregion from remote sensing images

**DOI:** 10.1038/s41597-023-02518-z

**Published:** 2023-09-09

**Authors:** Genyun Sun, Zheng Li, Aizhu Zhang, Xin Wang, Kai Yan, Xiuping Jia, Qinhuo Liu, Jing Li

**Affiliations:** 1 College of Oceanography and Space Informatics, China University of Petroleum (East China), Qingdao, 266580 China; 2 Laboratory for Marine Resources Qingdao National Laboratory for Marine Science and Technology, Qingdao, 266237 China; 3 Center for GeoData and Analysis, State Key Laboratory of Remote Sensing Science, Faculty of Geographical Science, Beijing Normal University, Beijing, 100875 China; 4 School of Engineering and Information Technology, The University of New South Wales, Canberra, ACT2600 Australia; 5 State Key Laboratory of Remote Sensing Science, Aerospace Information Research Institute, Chinese Academy of Sciences, Beijing, 100101 China

**Keywords:** Environmental sciences, Planetary science

## Abstract

High-resolution and multi-temporal impervious surface area maps are crucial for capturing rapidly developing urbanization patterns. However, the currently available relevant maps for the greater Mekong subregion suffer from coarse resolution and low accuracy. Addressing this issue, our study focuses on the development of accurate impervious surface area maps at 10-m resolution for this region for the period 2016–2022. To accomplish this, we present a new machine-learning framework implemented on the Google Earth Engine platform that merges Sentinel-1 Synthetic Aperture Radar images and Sentinel-2 Multispectral images to extract impervious surfaces. Furthermore, we also introduce a training sample migration strategy that eliminates the collection of additional training samples and automates multi-temporal impervious surface area mapping. Finally, we perform a quantitative assessment with validation samples interpreted from Google Earth. Results show that the overall accuracy and kappa coefficient of the final impervious surface area maps range from 92.75% to 92.93% and 0.854 to 0.857, respectively. This dataset provides comprehensive measurements of impervious surface coverage and configuration that will help to inform urban studies.

## Background & Summary

**Fig. 1:**
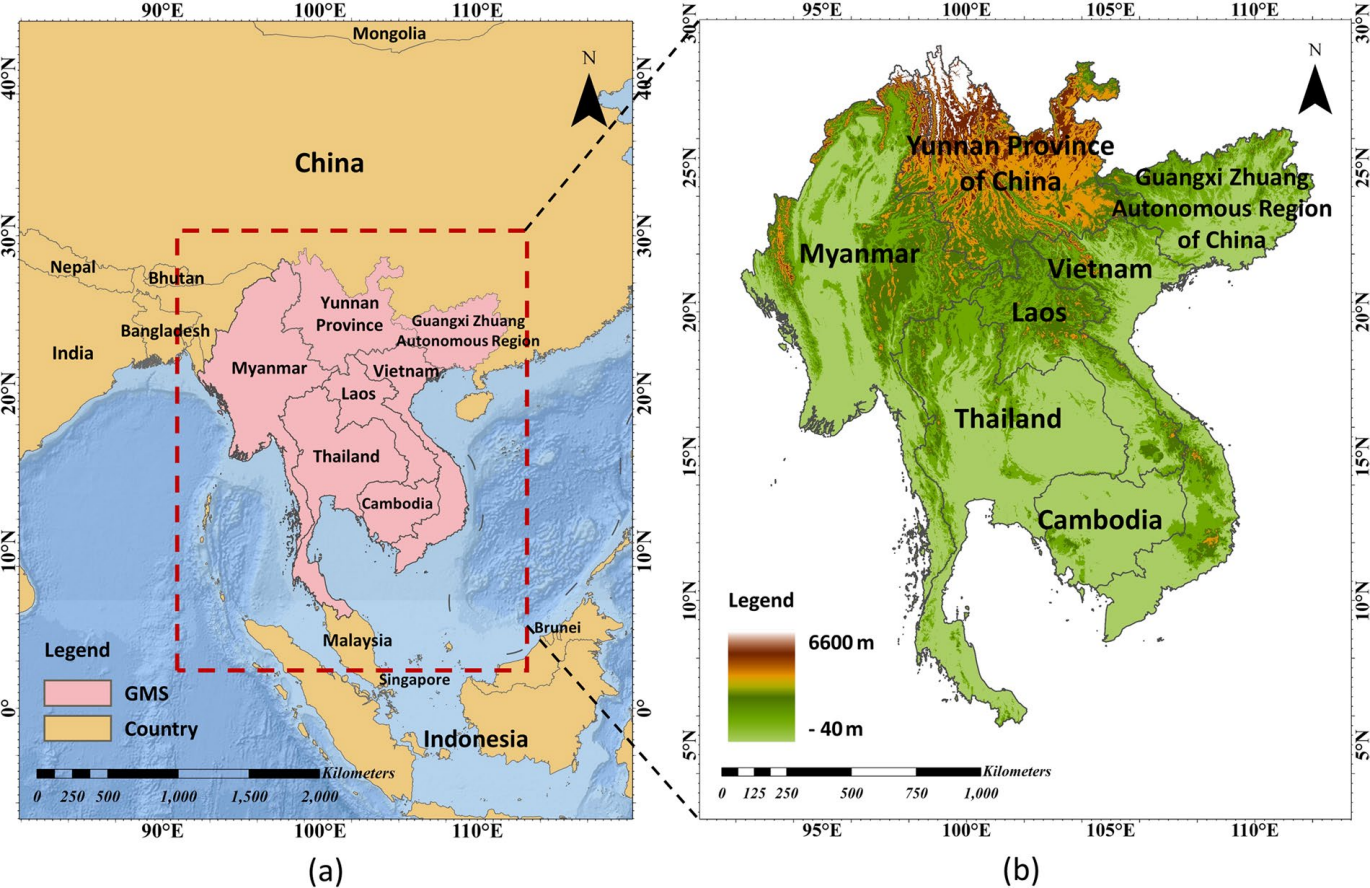
(**a**) The location of the Greater Mekong subregion (GMS), and (**b**) its elevation distribution. The imagery for the digital elevation model (DEM) was acquired from the Shuttle Radar Topography Mission (SRTM).

Continuous urbanization has been a widespread global trend in recent decades, especially in developing countries^1^. In this regard, the Greater Mekong Subregion (GMS), which covers Vietnam, Laos, Cambodia, Myanmar, and Thailand, as well as the Yunnan Province and Guangxi Zhuang Autonomous Region of China (Fig. 1), is undergoing an exceptionally rapid transition from rural to industrial societies^2^. Such dramatic urbanization has brought a cascade of environmental and socioeconomic challenges, including wetland degradation, forest destruction, and resource crises^3,4^. These challenges ultimately affect the sustainable development of society and human well-being^5–7^. Impervious surfaces refer to human-made materials that prevent water from infiltrating into soils, such as roads, roofs, and parking lots^8,9^. They are a sign of human activity and also serve as a critical indicator of the urbanization process^10,11^. The explicit spatial distribution of impervious surfaces offers a better insight for exploring the driving forces and characteristics of urbanization. Therefore, high-resolution and multi-temporal maps quantitatively depicting the dynamics of impervious surfaces in the GMS are increasingly crucial for effectively addressing the aforementioned problems^12–15^. Such datasets provide detailed information about anthropogenic change and are valuable for studying urban environment in fields of hydrology, ecology, geography, planning, etc.

Currently, satellite remote sensing offers unique support for regional and even global impervious surface area mapping with its scheduled revisits, extensive coverage, and comparative affordability^9^. Especially since 2008, when the entire Landsat archive became freely available, multispectral images (MSI) from this program have become the main data source for land cover change studies due to their long duration (nearly 40 years) and medium spatial resolution (30 m)^16–19^. Multi-temporal datasets such as GAIA^20^, GlobleLand30^21^, GISD^22^ and GISA^23^, which have been produced using Landsat data, have been instrumental in regional sustainable development studies. However, in the agriculture-dominated GMS, impervious surfaces tend to be small and patchy, often smaller than the minimum imaging unit of Landsat images. Even in urban cores with larger impervious surface areas, the heterogeneity of landscapes results in image pixels whose albedo is confused with other land covers. Therefore, maps based solely on Landsat data may not provide adequate guidance for finer-scale urban research.

The launch of the Sentinel-2 mission’s two satellites has led to the gradual development of advanced 10-m impervious surface area maps, such as FROM-GLC10^24^, GHS-S2^25^, Esri Land Cover^26^, and Dynamic World^27^. The improved resolution facilitates a deeper understanding of the coverage and configuration for impervious surfaces. However, samples sourced globally can be unsuitable for detecting changes to impervious surfaces within a particular region. Therefore, training with geographical expertise embedded or developing maps tailored to unique regions is more responsive and beneficial for all stakeholders. However, higher-resolution images often come at the expense of weaker spatial and temporal coverage, entailing higher collection and mapping costs. While these data are now commonly used for impervious surface monitoring in affluent large cities, they are not well established for global or regional scale impervious surface area mapping.

Another concern is the impact of the GMS’ cloudy and rainy climate on the availability of cloud-free satellite optical images, resulting in these datasets being unreliable here. This limitation has prompted numerous studies to explore the fusion of multi-source data to supplement optical-based impervious surface area mapping^28–32^. Among the sources of alternative data, synthetic aperture radar (SAR) data stand out for their ability to sense geometric structures and electrical properties, making them responsive to buildings^33,34^. Importantly, SAR data are unaffected by weather conditions, thus offering the potential to generate more actionable maps^35–37^. The Sentinel-1 mission, which provides C-band SAR images with comparable spatial resolution and revisit frequency as Sentinel-2, allows the creation of enhanced 10-m thematic maps. Leveraging the capabilities of cloud-based image processing, the Google Earth Engine (GEE) platform archives all Sentinel-1/2 images and hosts ample classification algorithms^38^, opening up the opportunity for fine-scale mapping and monitoring of land cover or impervious surface globally or regionally.

This paper develops a new framework using the GEE platform for merging time-series Sentinel-1 SAR and Sentinel-2 MSI data for multi-temporal impervious surface area mapping, producing a final 10-m impervious surface area map for GMS covering the period 2016–2022.

## Methods

**Fig. 2:**
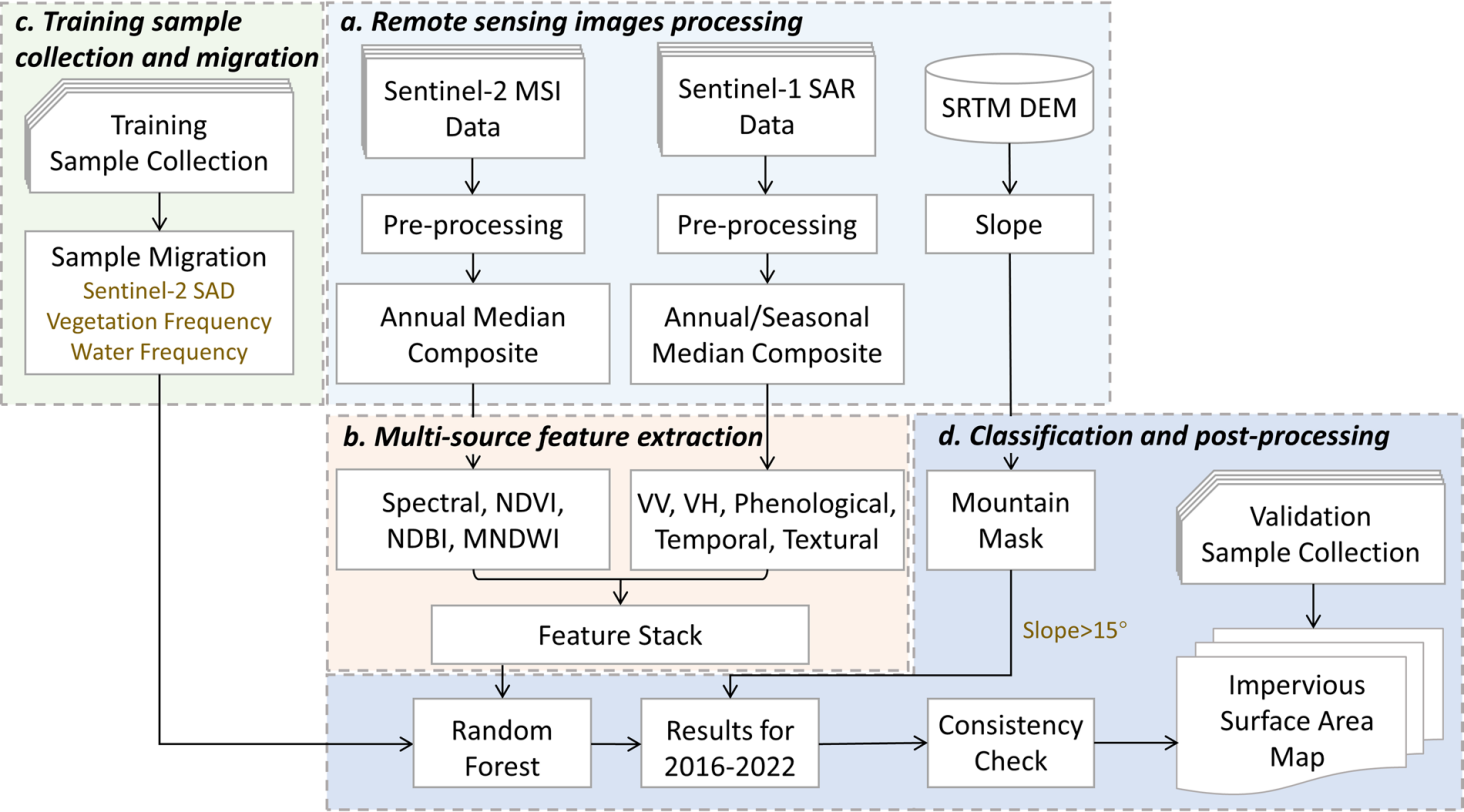
Workflow for developing multi-temporal impervious surface area maps. MSI: Multispectral Images; SAR: Synthetic Aperture Radar; SRTM: Shuttle Radar Topography Mission; DEM: Digital Elevation Model; SAD: Spectral Angular Distance; NDVI: Normalized Difference Vegetation Index; NDBI: Normalized Difference Built-up Index; MNDWI: modified Normalized Difference Water Index; VV: vertical-vertical band of Sentinel-1 images; VH: vertical-horizontal band of Sentinel-1 images.

Figure 2 summarizes our workflow for developing multi-temporal impervious surface area maps, including (a) remote sensing image processing, (b) multi-source feature extraction, (c) training sample collection and migration, and (d) classification and post-processing.

### Remote sensing images processing

We developed a GEE script to process online 173,247 Sentinel-2 MSI images and 46,856 Sentinel-1 SAR images covering the GMS.

The Sentinel-2 MSI L1C data, which represent top of atmosphere (TOA) reflectance, contain 13 bands with wavelengths ranging from 443 nm to 2190 nm and spatial resolution ranging from 10 m to 60 m. For our analysis, we used only blue, green, red, and NIR bands at 10-m spatial resolution and narrow NIR, Red Edge 1–3, and SWIR 1–2 bands at 20-m spatial resolution. These bands are labelled B2, B3, B4, B5, B6, B7, B8, B8A, B11, and B12. All 20-m bands were resampled to 10-m resolution adopting the nearest-neighbour interpolation method. Prioritizing data quality, we retained only the images with less than 50% cloud cover. Additionally, we removed their cloud-contaminated pixels using the Quality Assessment (QA60) band. To obtain representative data for each year, we selected the pixel with the median value from multiple images taken at the same location within the same year. This operation of annual median composition removes extremely dark or bright pixels that may be caused by scene-specific illumination conditions^24^.

From Sentinel-1 products, we selected the Level-1 Ground Range Detected (GRD) scenes with vertical-vertical (VV) and vertical-horizontal (VH) bands in Interferometric Wide (IW) swath mode operated in an “ascending” or “descending” orbit. Each GRD image archived on the GEE platform has been pre-processed by the Sentinel-1 Toolbox, including thermal noise removal, radiometric calibration, and terrain correction, with a final conversion to the backscatter coefficient (σ°) in decibels (dB). To mitigate the influence of layover, speckle noise, and shadows, we will be using mean annual values for VV and VH in further calculations^39^.

### Multi-source feature extraction

We calculated three spectral indices, namely the Normalized Difference Vegetation Index (NDVI), Normalized Difference Built-up Index (NDBI), and modified Normalized Difference Water Index (mNDWI), to improve the distinction between vegetation, built-up areas, and water, which were the dominant land covers in the GMS. Their calculations are shown in Eq. 1 to Eq. 3:

1 $$NDVI=\frac{NIR-Red}{NIR+Red}$$

2 $$NDBI=\frac{SWIR1-NIR}{SWIR1+NIR}$$

3 $$mNDVI=\frac{Green-SWIR1}{Green+SWIR1}$$

where Green is the green band (wavelength: 543–578 nm), Red is the red band (wavelength: 650–680 nm), NIR is the near infra-red band (wavelength: 785–900 nm) and SWIR1 is the short-wave infrared 1 (wavelength: 1565–1655 nm).

We then derived the annual standard deviation of the VV and VH images from all GRD images available for each year to capture the temporal metrics of objects. Moreover, with reference to the climatic conditions in the GMS, we divided the annual VV and VH time-series images into four groups and performed intra-group mean compositions, yielding eight phenological backscattering metrics for each year. Since texture features highlight the local spatial characteristics of different land covers^40^, we calculated five texture variables for the VV and VH images each year using the gray-level co-occurrence matrix (GLCM). These texture variables included angular second moment (ASM), entropy (ENT), inverse difference moment (IDM), correlation (CORR), and sum average (SAVG). Previous studies have demonstrated the effectiveness of these variables in describing the texture of different urban land covers^41,42^. The texture features were calculated from the average of the directional bands within a 7 × 7 window. All these features, along with the annual composite optical and backscattering bands, were used as input to the classifier (Table 1).

**Table 1:** Input features for the classification.

Data source	Input features	Dimension
Sentinel-2 MSI	Spectral features: median composition of B2, B3, B4, B5, B6, B7, B8, B8A, B11, B12	10
	Normalized indices: NDVI, NDBI, MNDWI	3
Sentinel-1 SAR	Backscattering features: annual mean of VV, VH time-series images	2
	Phenological features: seasonal mean of VV, VH time-series images	8
	Temporal features: standard deviation of VH, VV	2
	Textural features: ASM, ENT, IDM, CORR, SAVG of each annual mean of VV and VH	10

See text for the meaning of all acronyms.

### Training sample collection and migration

The training samples were visually interpreted primarily based on the annual median composite Sentinel-2 images. Supplementary information from Google Earth images was also used. We adopted the clustered sampling technique^43^ to randomly allocate 2990 sample polygons across the GMS. Autocorrelation was attenuated by manipulating the number (about 10) and distance (>30 m) of samples within a polygon. Altogether, 9707 impervious surface samples and 18310 non-impervious surface samples were obtained for 2016. The impervious surface samples contained various features, such as buildings, roads, etc., while the non-impervious surface samples consisted of vegetation, water, and soil.

**Fig. 3:**
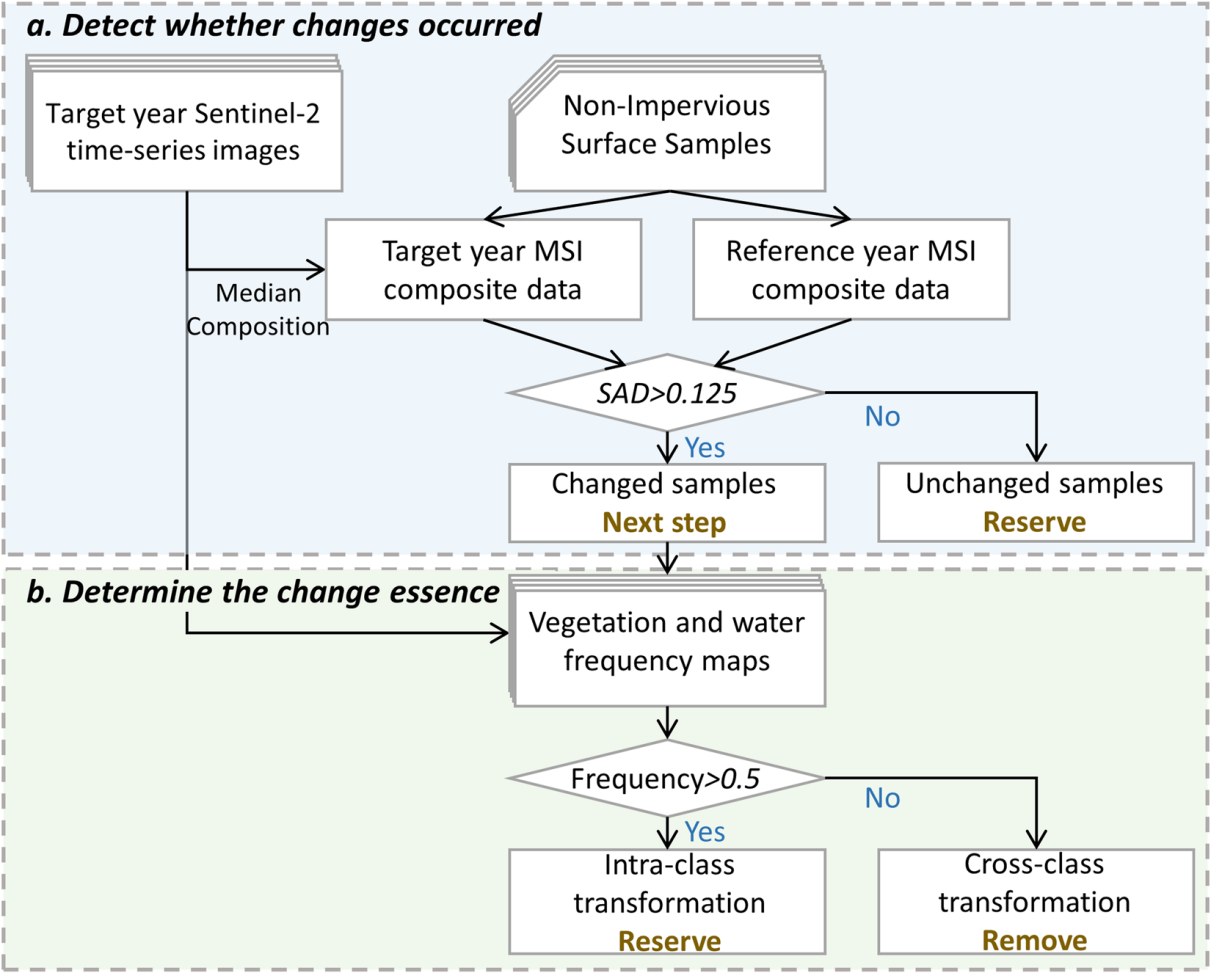
Flowchart of the proposed automatic training sample migration method.

Next, a sample migration method was applied to eliminate the redundant collection of ground truth for multi-temporal mapping (Fig. 3). This method assumes that the transformations from natural surface to impervious surface are irreversible over a short time^44^, implying that land cover changes occur primarily in non-impervious surface samples. We split the changed samples into two groups based on whether the label is affected, including intra-class transformations within non-impervious surfaces and cross-class transformations from non-impervious surfaces to impervious surfaces. In the context of binary classification of impervious surfaces, intra-class transformations, mostly involving vegetation, water, and soil, do not interfere with the results. Thus, the proposed method is designed to remove samples that have undergone cross-class transformations in two steps.

The spectral angular distance (SAD) was first applied to detect whether the land cover of the non-impervious surface sample had transformed, as in Eq. 4:

4 $$SAD=co{s}^{-1}\left(\frac{{X}_{r}^{T}{X}_{t}}{\sqrt{{X}_{r}^{T}{X}_{r}}\sqrt{{X}_{t}^{T}{X}_{t}}}\right)$$

where *X*_*r*_ are the reference spectra, and *X*_*t*_ are the target spectra at year t. Here, the spectra extracted from the beginning year’s Sentinel-2 MSI median composite data are considered the reference spectra, while those of the other years are considered the target spectra.

Afterward, a thresholding of 0.125 was experimentally set to determine the samples with a high probability of experiencing land cover changes. The unchanged samples were reserved as non-impervious surface training samples for the target year. For the changed samples, NDVI and mNDWI were utilized to count the samples’ vegetation and water frequencies in the target year, as in Eqs. 5, 6. Samples with any frequency greater than 0.5, indicating a higher likelihood of being non-impervious in the target year, were considered to have undergone only intra-class transformations and were consequently retained. The remaining samples were removed from the training set.

5 $$Vegetation\;Frequency={N}_{NDVI > 0.25}/{N}_{Total}$$

6 $$Water\;Frequency={N}_{mNDWI > 0.12}/{N}_{Total}$$

where *N*_*NDVI*>0.25_ and *N*_*NmNDWI>*0.12_ denote the number of times that a sample meets the subscript condition in the target year, and *N*_*Total*_ is the times this pixel was retained in that year. We tested a sequence of thresholds ranging from 0.1 to 0.5, with a 0.01 step, to determine the optimal thresholds for these two equations.

### Classification and post-processing

Random forest (RF) is an ensemble classifier consisting of numerous decision trees, with each tree growing from randomly selected samples and features based on the bootstrap aggregation technique. The final result depends on the majority vote of all decision trees. RF has two primary parameters: the number of trees (Ntree) and the number of variables split at each tree node (Mtry). In view of the computational efficiency and on a trial-and-error basis, Ntree was set as 500, Mtry was set as the square root of the number of input variables. For each year, a separate RF classifier was trained using migrated samples to extract impervious surfaces. The computational power necessary for this task was supported by the GEE platform.

In mountainous areas, the spectra and backscatter of bare rock closely resemble those of urban buildings. Consequently, pixel-level classifications often confuse these two land cover types. To address this issue, we calculated the slope from the DEM data and labelled the classified pixels with a slope greater than 15° as non-impervious surfaces. Additionally, we applied a median spatial filter with 3 × 3 windows to reduce the effect of “salt and pepper” noise.

In time-continuous mapping domains, misclassification can potentially lead to unreasonable sequences of results. To mitigate this problem, we first employed temporal filtering. For each pixel, a 1-D filter was applied to iterate through its time sequence, and the label of the filter centre was modified via majority voting. Then, we gradually increased the filter size and repeated this procedure until all the labels in the sequence no longer changed. Following this, we performed a consistency check to ensure the irreversibility of impervious surfaces: (1) if the next three years were all non-impervious, then the label for that year was set to non-impervious surface; (2) if the previous three years were all impervious, then the label for that year was set to impervious surface.

## Data Records

**Fig. 4:**
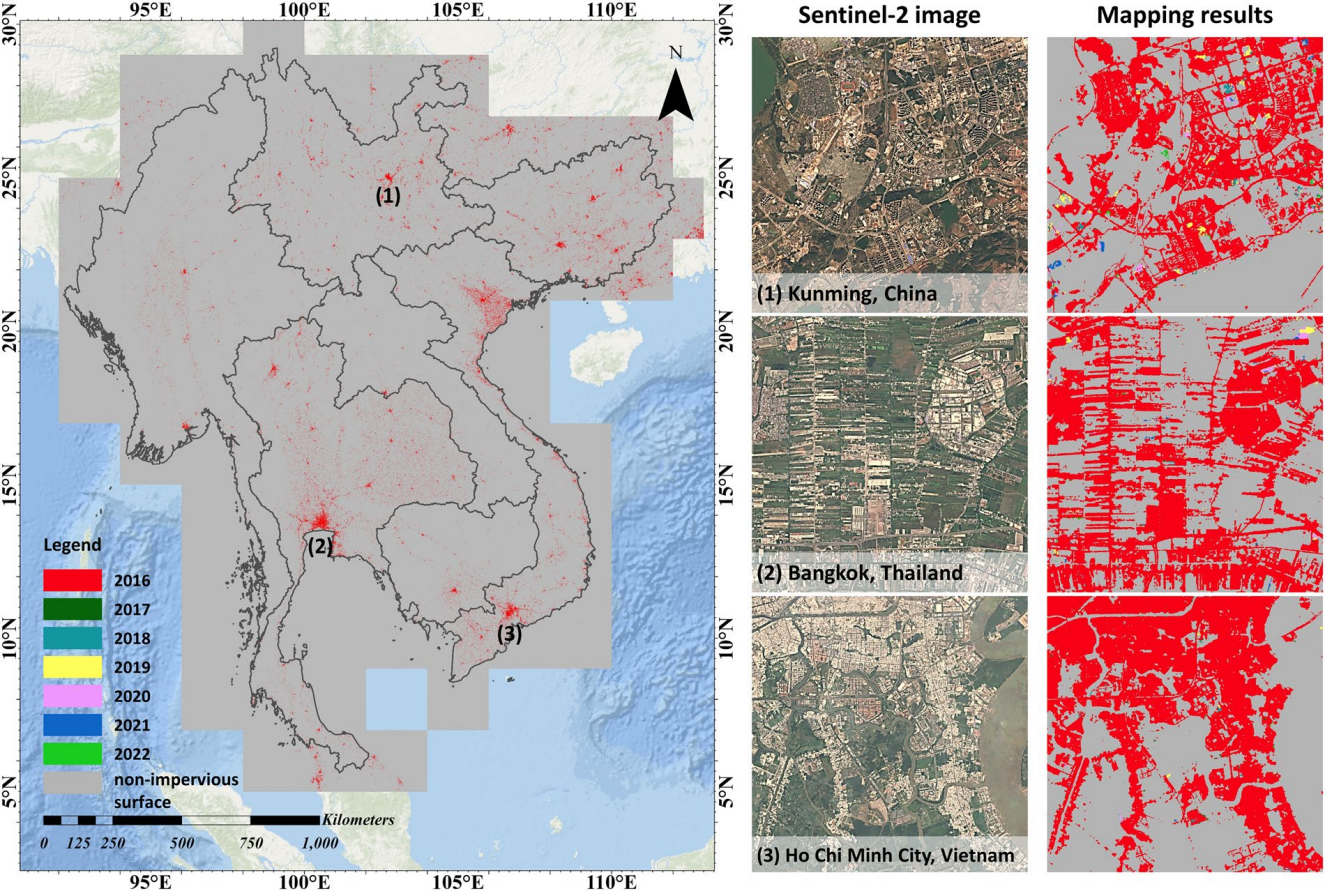
Sample results of the impervious surface area map. The left panel presents its thumbnail, while the right panel presents the Sentinel-2 images used for mapping and their corresponding results.

**Fig. 5:**
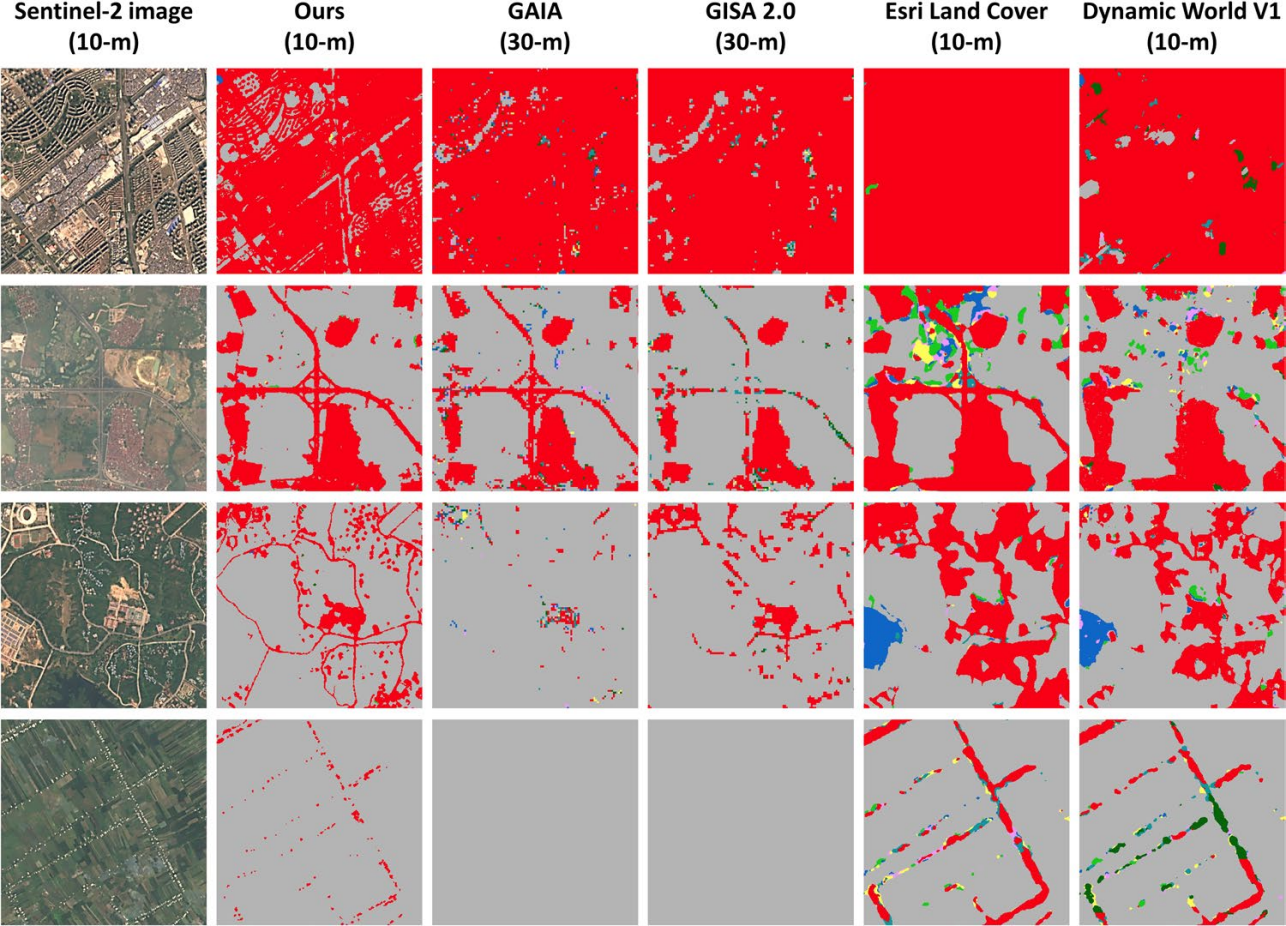
Comparison of the impervious surface area map with other datasets. The first column presents Sentinel-2 images in 2022, the second column presents our impervious surface area map, and other the columns present some examples of existing multi-temporal maps. The legend for this figure is the same as for Fig. 4.

We have made the final impervious surface area maps for the GMS publicly available in the figshare repository (10.6084/m9.figshare.21836196.v3)^45^. The dataset includes three sets of files: (1) the map data in GEOTIFF format, (2) the distribution of the tiles in shapefile format, and (3) the thumbnails of the dataset in JPEG format. This dataset can be viewed and processed with geographic information and remote sensing software, programming packages, and cloud platforms such as ArcGIS, QGIS, ENVI, GDAL, and GEE. The details of the metadata are given in Table 2, some samples of the impervious surface area map are shown in Fig. 4, and the comparison with other datasets is shown in Fig. 5.

**Table 2:** The details of the impervious surface area map.

Spatial Extent	Top: 31°N, Bottom: 5°N, Left: 92°E, Right: 114°E
Cell Size	10 meters × 10 meters
Number of Bands	1 band named “b1”
Storage format	111 tiles (2° × 2°) in GEOTIFF format
Coordinate System	WGS 1984 (EPSG: 4326)
Pixel type	16 Bit unsigned short
Pixel values	0: non-impervious surfaces,
	2016: existing impervious surfaces in 2016 and before,
	2017: newly expanded impervious surfaces in 2017
	2018: newly expanded impervious surfaces in 2018
	…
	2022: newly expanded impervious surfaces in 2022

## Technical Validation

**Fig. 6:**
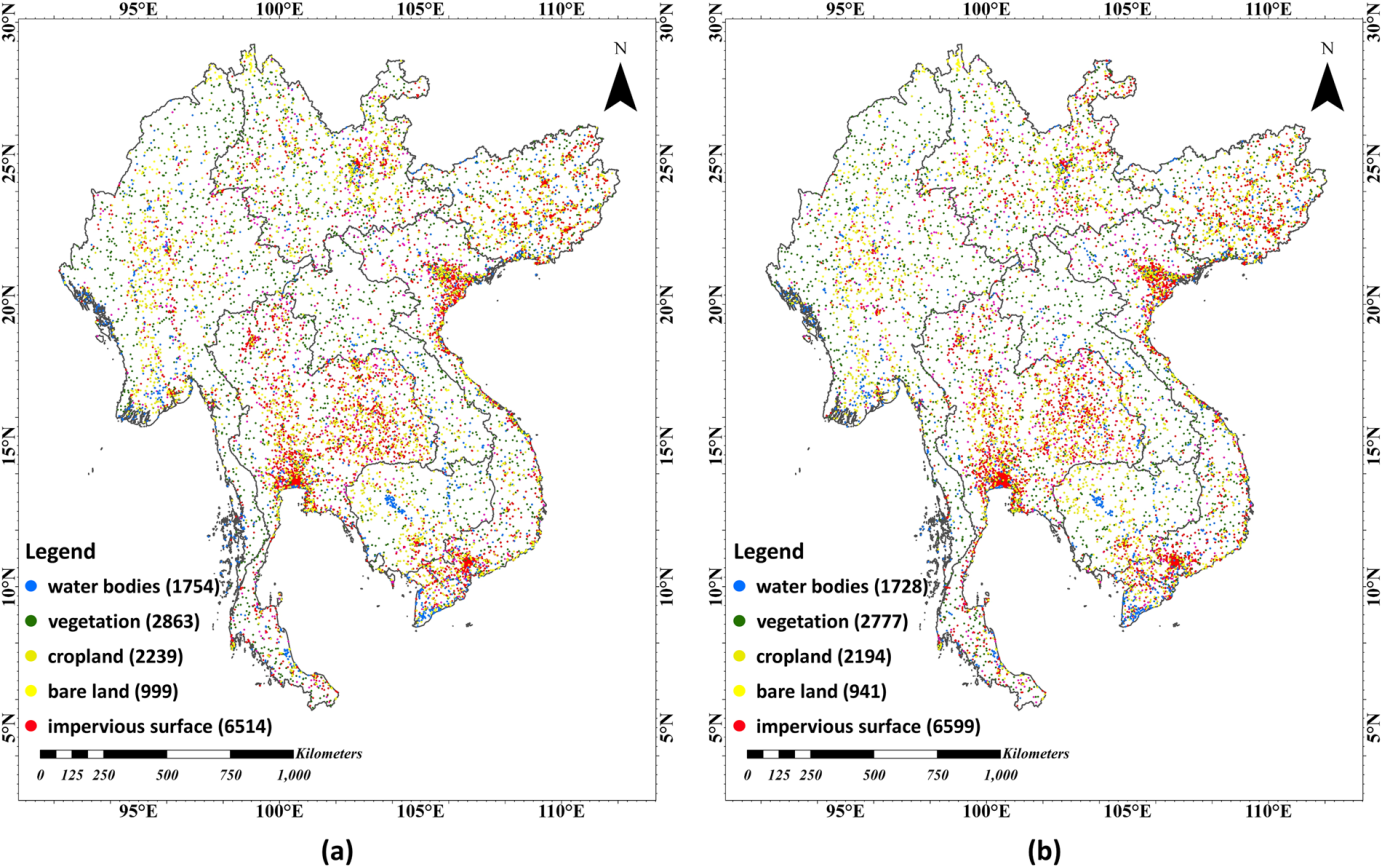
Size and distribution of the validation samples, (**a**) validation samples for 2016, (**b**) validation samples for 2022.

We applied a stratified random sampling strategy for technical validation. The subgroups of stratification include water bodies, vegetation (non-crop), cropland, bare land, and impervious surfaces. To assess the temporal component of the dataset, samples were collected from 2016 and 2022. Finally, Google Earth images were used to visually interpret the samples. The size and distribution of the validation samples are depicted in Fig. 6.

Then, four accuracy metrics, including the overall accuracy (OA), user’s accuracy (UA), producer’s accuracy (PA), and kappa coefficient (Kappa), were calculated based on the validation samples. The complete validation results of the impervious surface area map for the entire GMS are shown in Table 3. Additionally, the dataset in urban areas and in suburban/rural areas was validated individually using subsets of validation samples. The OA is 93.69% and 93.66% for urban areas and 91.12% and 90.96% for suburban/rural areas, respectively.

**Table 3:** Accuracy assessment based on validation samples. See text for the meaning of all acronyms.

	OA (%)	Kappa	impervious surface		non-impervious surface	
			UA (%)	PA (%)	UA (%)	PA (%)
2016	92.93	0.857	94.41	89.71	91.80	95.60
2022	92.75	0.854	94.96	89.06	91.06	95.93

## Usage Notes

With this dataset, researchers could describe the spatial distribution of impervious surfaces in the GMS, such as coverage and configuration. The map’s temporal continuity further enables the calculation of dynamic metrics of urban expansion, such as speed, intensity, and orientation, etc. However, uncertainties still persist within this dataset. The presence of mixed pixels in Sentinel images poses challenges for accurately recognizing meter and sub-meter targets. Therefore, when it comes to micro-analysis, we encourage orthophotos to be used. The overlays of our dataset with other land covers’ datasets, for example, vegetation and water maps, allows exploring more sustainable urban planning solutions. But the temporal matching of different datasets needs to be carefully considered. Given the build rate of impervious surfaces, we employed annual composite images. This is unsuitable for shorter-term dynamic analysis. The above programs can be implemented with geographic information and remote sensing software, programming packages, and cloud platforms such as ArcGIS, QGIS, ENVI, GDAL, and GEE.

## Data Availability

Every step from obtaining Sentinel data to processing images into impervious surface temporal data was done in Google Earth Engine. All steps, including image processing, sample migration, and image classification have been made publicly available in the GEE code snippet (https://code.earthengine.google.com/74d0845d708a01fde1484c30ca73cc72). The collected training and validation samples have been archived in the same code snippet.
